# Quantitative map of multiple auditory cortical regions with a stereotaxic fine-scale atlas of the mouse brain

**DOI:** 10.1038/srep22315

**Published:** 2016-02-29

**Authors:** Hiroaki Tsukano, Masao Horie, Ryuichi Hishida, Kuniyuki Takahashi, Hirohide Takebayashi, Katsuei Shibuki

**Affiliations:** 1 Department of Neurophysiology, Brain Research Institute, Niigata University, 1-757 Asahimachi-dori, Chuo-ku, Niigata 951-8585, Japan; 2 Division of Neurobiology and Anatomy, Graduate School of Medicine and Dental Sciences, Niigata University, 1-757 Asahimachi-dori, Chuo-ku, Niigata 951-8510, Japan; 3 Department of Otolaryngology, Graduate School of Medicine and Dental Sciences, Niigata University, 1-757 Asahimachi-dori, Chuo-ku, Niigata 951-8510, Japan

## Abstract

Optical imaging studies have recently revealed the presence of multiple auditory cortical regions in the mouse brain. We have previously demonstrated, using flavoprotein fluorescence imaging, at least six regions in the mouse auditory cortex, including the anterior auditory field (AAF), primary auditory cortex (AI), the secondary auditory field (AII), dorsoanterior field (DA), dorsomedial field (DM), and dorsoposterior field (DP). While multiple regions in the visual cortex and somatosensory cortex have been annotated and consolidated in recent brain atlases, the multiple auditory cortical regions have not yet been presented from a coronal view. In the current study, we obtained regional coordinates of the six auditory cortical regions of the C57BL/6 mouse brain and illustrated these regions on template coronal brain slices. These results should reinforce the existing mouse brain atlases and support future studies in the auditory cortex.

The auditory cortex is composed of multiple regions to realize hierarchical processing for complex auditory perception^1^,^2^,^3^. Previous studies using optical imaging have demonstrated at least six regions in the mouse auditory cortex^4^,^5^, including four frequency-organized regions — the anterior auditory field (AAF), primary auditory cortex (AI), secondary auditory field (AII), and dorsomedial field (DM) — and two frequency-unorganized regions — the dorsoanterior field (DA) and dorsoposterior field (DP) (Fig. 1a). Delineation of this functional map is consistent with differences in molecular distribution^5^,^6^ and projections from the auditory thalamus, the medial geniculate body (MGB)^5^,^7^,^8^.

Optical imaging has been a useful tool for fine-grained mapping in mouse sensory cortices^9^,^10^,^11^,^12^,^13^,^14^. In mapping the mouse auditory cortex, flavoprotein fluorescence imaging which reveals intrinsic signals coupled with aerobic metabolism^15^, or detection of fluorescence in mice expressing the calcium indicator protein GCaMP3^16^, has uncovered many important structures, for example the frequency organization in AII^16^,^17^, a new frequency gradient in the AI^4^,^5^,^16^, and a new region DM^4^,^5^. Thus, endogenous fluorophore imaging is a powerful means of delineating small cortical regions with a width of ~300 μm as it avoids the inhomogeneous staining associated with the use of exogenous chemical fluorescent dyes^5^,^16^.

World-wide efforts are developing precise, reliable, useful references of the mouse brain. Precise brain atlases illustrating coronal sections are useful for identifying brain regions in slice sections, and encourage the use of common nomenclature in neuroscience research^18^,^19^. The long-standing brain atlas published by Paxinos and Franklin was established according to chemoarchitectonic patterns^19^; it has become the standard reference for the anatomy of the C57BL/6 mouse brain, and was reconstructed into a 3-D atlas^20^. This atlas is helpful because it covers all brain regions from the pons to the neocortex, and users can recognize various brain regions at a glance on a macroscopic scale. Furthermore, recent brain mapping projects performed by the Allen Institute have provided a wide range of information about the mouse brain with an elaborate segmentation^18^. There, a visual cortex map with at least 10 higher-order regions elucidated by physiological^9^,^10^,^12^ and neurotracing studies^21^ and a comprehensive body surface map of the somatosensory cortex have been portrayed. However, a fine auditory cortical map with multiple auditory regions is not currently available.

In the current study, we identified stereotaxic coordinates of the six auditory cortical regions of the C57BL/6 mouse by flavoprotein fluorescence imaging, and denoted their position in coronal brain slices. The mouse model is widely used in neuroscience research due to advantages in its applicability of various experimental tools, genetic tractability^22^,^23^, and lissencephalic cortex^24^. The physiological identification of the auditory cortical regions will contribute to establishing a standard mouse brain database.

## Results

### Identification of multiple regions in the mouse auditory cortex using flavoprotein fluorescence imaging

**Figure 1:**
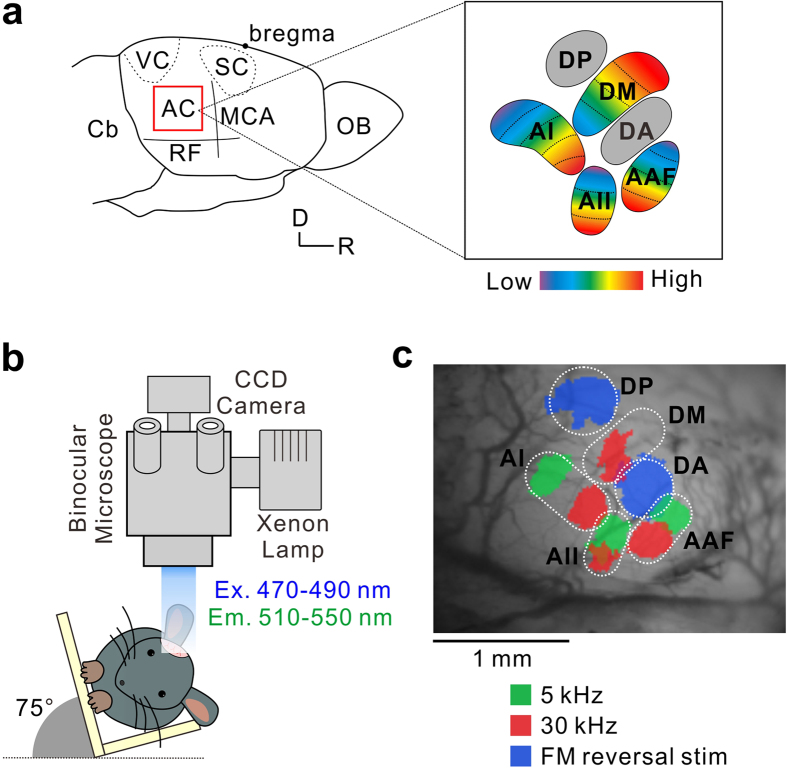
Map of the auditory cortex visualized using flavoprotein fluorescence imaging. (**a**) Schematic drawing of the right auditory cortex in mice. AAF, anterior auditory field; AI, primary auditory cortex; AC, auditory cortex; AII, secondary auditory field; Cb, cerebellum; DA, dorsoanterior field; DM, dorsomedial field; DP, dorsoposterior field; MCA, medial cerebral artery; OB, olfactory bulb; RF, rhinal fissure; SC, somatosensory cortex; VC, visual cortex. D, dorsal; R, rostral. (**b**) An illustration of the experimental setup for flavoprotein fluorescence imaging. Imaging was performed with the head rotated ~75° to position the auditory cortex perpendicular to the microscope. Ex, excitation; Em, emission. (**c**) A typical image of the right auditory cortex map visualized using flavoprotein fluorescence imaging. D, dorsal; R, rostral.

We used flavoprotein fluorescence imaging^15^ to reveal tonal responses in the auditory cortex of the C57BL/6 mouse brain. Anesthetized mice were fixed with the head rotated about 75° to bring the surface of the right auditory cortex to the microscope (Fig. 1b). Precise locations of the six regions were identified in the brain surface view (Fig. 1c). We presented 5- and 30-kHz tones to mice to generate tonotopic shifts in the AAF, AI, and AII, that were clearly distinguishable in these regions^4^,^5^,^16^. In addition, the tonotopic gradients of these regions are known to be arranged in a logarithmic manner in mammals including mice^4^,^5^,^16^,^25^, rats^26^,^27^, and cats^28^,^29^, therefore tonal stimuli over 30 kHz elicited weak responses in almost the same locations as those to 30 kHz (Supplemental Fig. S1). In the DM, responses to low frequency sounds around 5–20 kHz were weak and diffuse^4^,^5^,^16^. The 20- to 30-kHz tones elicited responses in almost the same region close to the ventral border of the AI^4^,^5^. A dorsal shift in tonotopy was observed using tones at 30 kHz up to about 60 kHz in the DM^4^,^5^,^16^. Therefore, we used 30-kHz and 60-kHz tones to obtain response shifts in the DM. To visualize neural responses in the frequency-unorganized regions of the DA and DP, which were localized on both sides of the DM, we used frequency modulation (FM) directional reverse stimuli^5^,^7^,^30^.

### Identification of the stereotaxic coordinates of auditory cortical regions using flavoprotein fluorescence imaging

**Figure 2:**
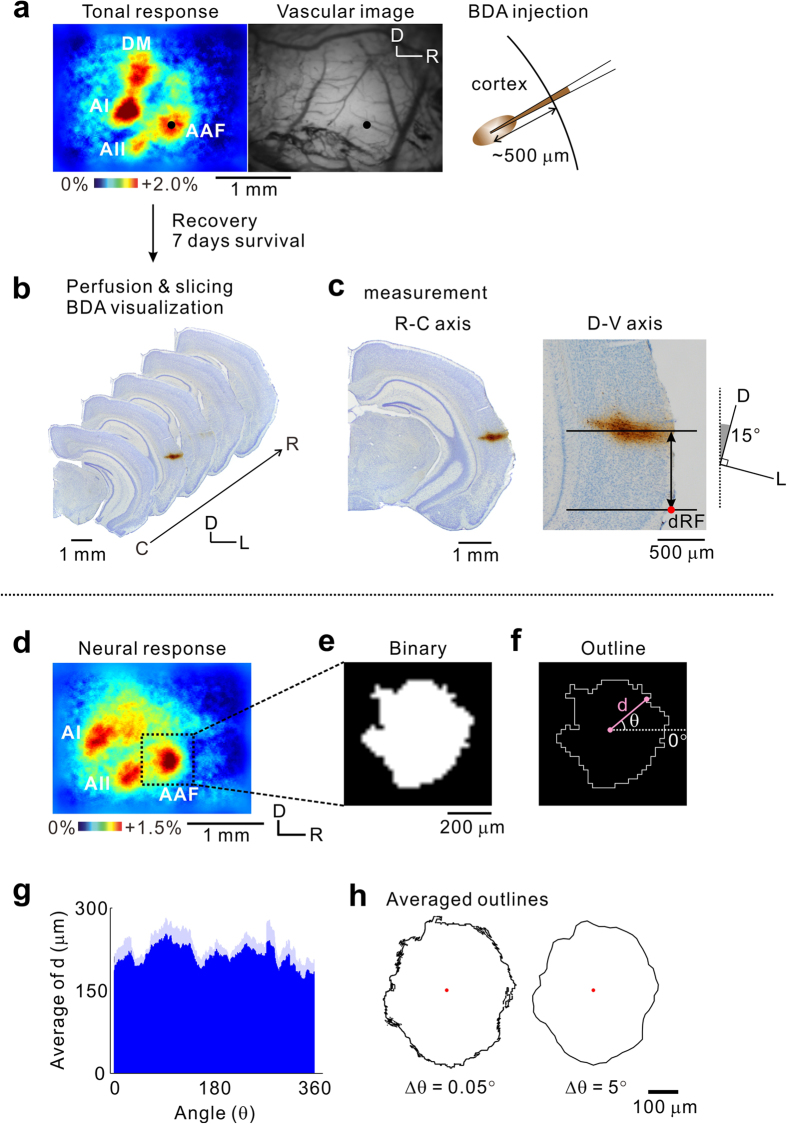
Acquisition of regional coordinates using imaging. (**a**) A tonal response to a 30-kHz tone (left). The vascular image on the cortical surface in the same mouse (right). A glass pipette filled with BDA was inserted into the center of the target region (black spots), and BDA was slowly injected by iontophoresis at a depth of 500 μm from the surface. (**b**) Visualization of BDA. Seven days after the injection, consecutive brain slices were prepared and BDA was visualized. (**c**) Measurement of the injection site locations. The brain slice with the strongest BDA staining was selected. The rostrocaudal coordinate of the slice was judged by the coronal view in reference to those showed in the Paxinos and Franklin brain atlas^20^. The dorsoventral coordinate of the injection site was obtained as a distance between the dorsal edge of the rhinal fissure and the line penetrating the center of the BDA-stained area. This measurement was performed after rotating the image by 15°. dRF, dorsal tip of the rhinal fissure. (**d**) A tonal response to a 5-kHz tone. (**e**) The image of the tonal response of the AAF, trimmed from the original image of (**d**). The image was converted into binary by setting the threshold to 60% of the peak value in the AAF response. (**f**) Edge detection from the binary image. The edge was obtained by applying a Sobel filter to the binary image after the pixel density was increased 2,500 times. (**g**) The distance between the center and each pixel composing the outline, indicated by d in (**f**). d values were obtained every 0.05°. The distance value was averaged by angle across five animals. Dark blue, mean data; light blue, standard error of the mean. (**h**) Averaged outlines. After the averaged outline of the 5-kHz area in the AAF was obtained (left), the outline was downsampled 1/100 to a final angular interval of 5°, to smoothen the outline (right). The red plot indicates the center of the outline. Experiments shown in (**a–c**) and (**d–h**) were conducted using different animal groups. C, caudal; D, dorsal; D-V, dorsoventral; L, lateral; R, rostral; R-C, rostrocaudal.

In order to histologically verify physiologically-mapped cortical fields, we injected biotinylated dextran amine (BDA) to mark the auditory cortical regions which were identified using flavoprotein fluorescence imaging (Fig. 2). This procedure allowed the same regions to be identified after the brain slices were prepared. BDA was used as it can be injected iontophoretically *in vivo* and observed in coronal brain sections. We injected BDA into the center of neural responses (Fig. 2a), and visualized it in consecutive coronal slices. Injected BDA was clearly tracked as a single, thin trajectory^8^ (Fig. 2b).

We recorded the locations of all the injection sites as rostrocaudal and dorsoventral coordinates, based on the position of the bregma and rhinal fissure (Fig. 2c). The rostrocaudal distance from the bregma was determined, comparing the coronal view of the Nissl-stained slice with the corresponding slice in the Paxinos and Franklin atlas^19^. This method provides reliable standard values for the distance from the bregma in C57BL/6 mice^6^,^20^,^31^,^32^. We evaluated the dorsoventral level of the injection site by measuring the distance between the dorsal edge of the rhinal fissure and the line penetrating the center of the injection site on the images rotated clockwise by 15° (Fig. 2c, right panel). This adjusted the observation view of the auditory cortex to match that seen under the microscope (Fig. 1b). All the coordinates of the injection sites are summarized in Table 1.

Next, we obtained information about regional sizes. Regional boundaries were obtained from tonal responses revealed using flavoprotein fluorescence imaging (Fig. 2d–h) as performed by Kalatsky and Stryker in the mouse visual cortex^33^. Response regions were identified as regions with pixels exhibiting fractional fluorescence changes >60% of the peak response amplitudes. A threshold of 60% was selected as it avoided gaps or overlaps between adjacent regions. The regional outlines were averaged across animals by calculating the distance between the center coordinate and each pixel composing the outline (Fig. 2f, letter d). The distances were averaged across animals by angle (Fig. 2g). We represented these data on a 2-D plot (Fig. 2h, left panel). Finally, the outline was downsampled so as to average the points within every 5° angle (Fig. 2h, right panel). We applied these procedures to other tonal responses and obtained 10 tonal response outlines of the AAF to 5 kHz and 30 kHz, the AI to 5 kHz and 30 kHz, the AII to 5 kHz and 30 kHz, the DA, the DM to 30 kHz and 60 kHz, and the DP, which were averaged in five animals each.

**Figure 3:**
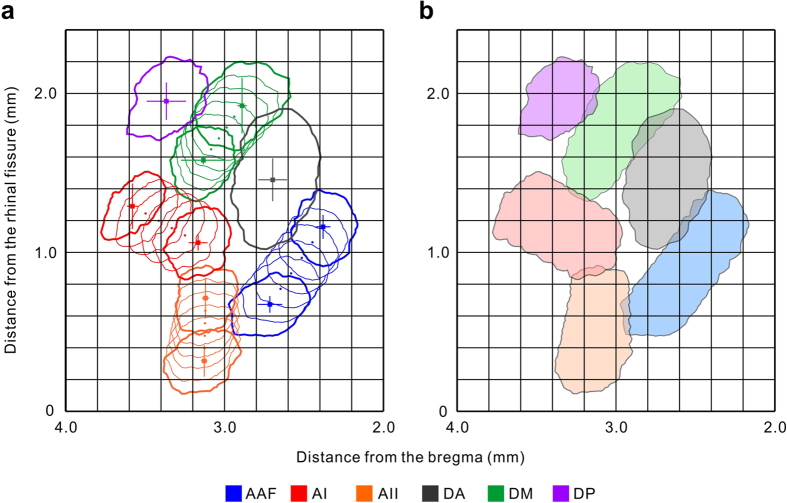
Reconstructed auditory cortical surface map. (**a**) Distribution of injection sites and outlines of responses on the coordinates. The 10 injection sites and 10 outlines obtained from the real data were demonstrated by square plots and bold lines respectively. The additional data to bridge the gap between the low and high-frequency areas were drawn by thin lines, the centers of which are shown by circular plots. Error bars indicate standard error of the mean. (**b**) The auditory cortical map where small outlines were combined.

We placed these outlines on the coordinates, so that the center of the outline was adjusted to the coordinates of the injection sites (Fig. 3a, bold line). The additional intermediate outlines were calculated such that they gradually changed shape from the outline of the 5-kHz area towards that of the 30-kHz area (or from the 30-kHz area towards the 60-kHz area in the DM) (Fig. 3a). The whole regional outline was smoothly delineated by combining the six small outlines (Fig. 3b). Overall, using the injection site coordinates (Fig. 2c, Table 1) and the regional outlines (Fig. 2h), we reconstructed an auditory cortical map that was similar to the neural responses observed using *in vivo* flavoprotein fluorescence imaging (Fig. 3b). The reconstructed regions are laid in an orderly manner without unnecessary gaps, which affirms the reliability of our methods and criteria in marking auditory cortical regions, obtaining the coordinates of the injection sites and converting the response image into a binary scheme.

### Creating a coronal atlas of the mouse auditory cortex

**Figure 4:**
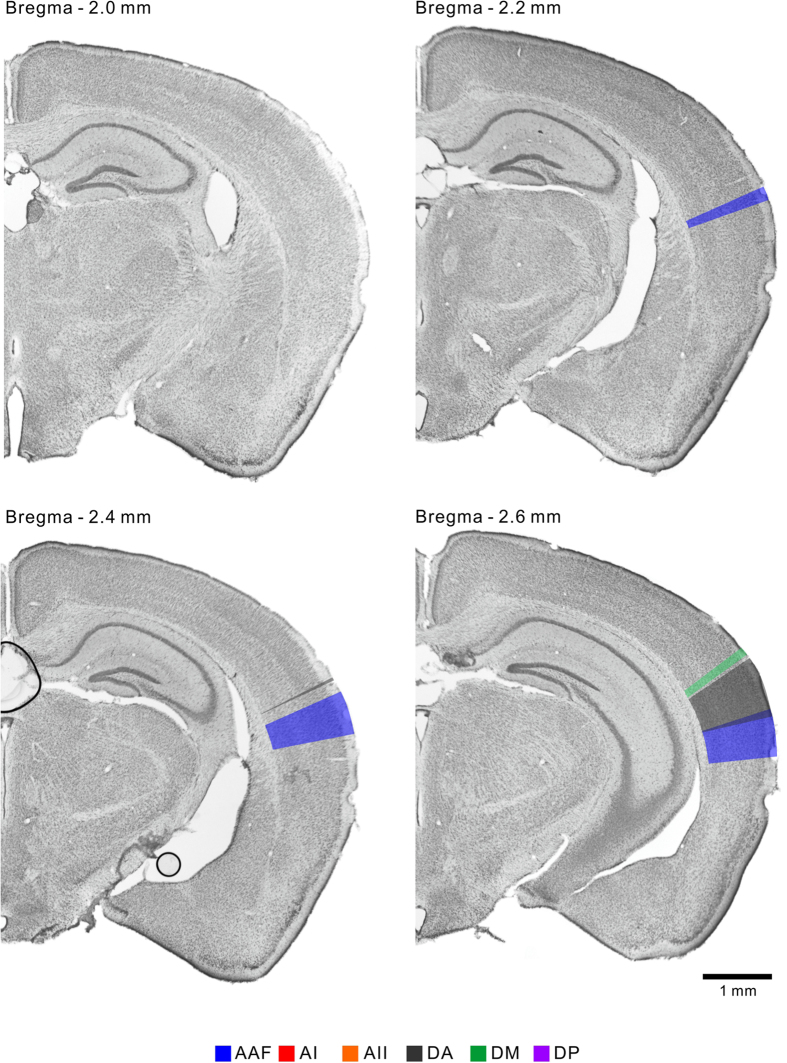
Coronal representation of the auditory cortical regions. The auditory cortical regions were represented on the template coronal slices from 2.0–2.6 mm inclusive, posterior to the bregma. All the images represent brain slices of the right hemisphere. Top, dorsal; right, lateral.

**Figure 5:**
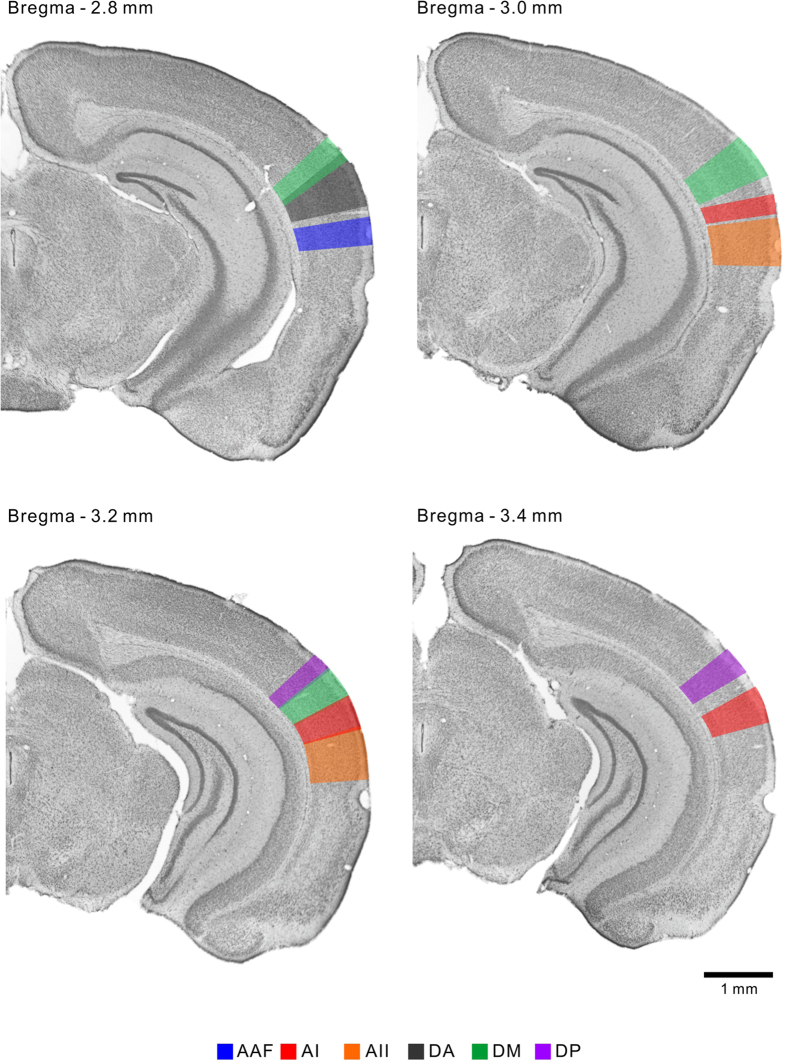
Coronal representation of the auditory cortical regions. The auditory cortical regions were represented on the template coronal slices from 2.8–3.4 mm inclusive, posterior to the bregma. All the images represent brain slices of the right hemisphere. Top, dorsal; right, lateral.

**Figure 6:**
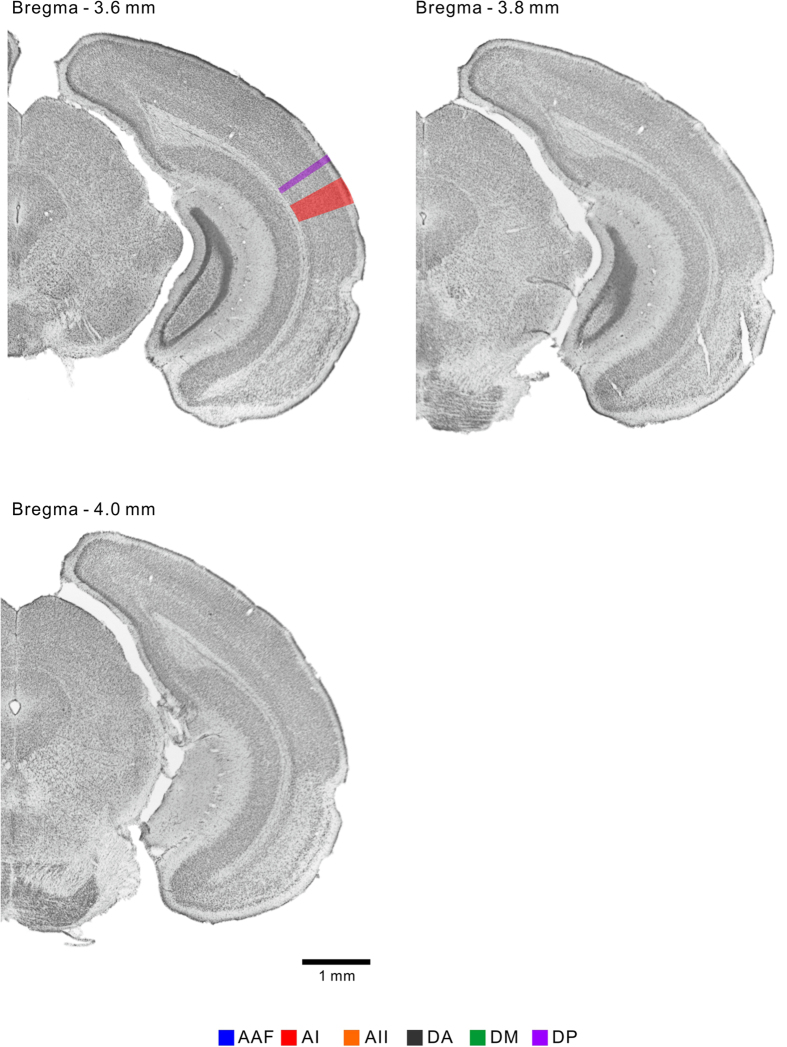
Coronal representation of the auditory cortical regions. The auditory cortical regions were represented on the template coronal slices from 3.6–4.0 mm inclusive posterior to the bregma. All the images represent brain slices of the right hemisphere. Top, dorsal; right, lateral.

Based on the reconstructed map, we delineated the six regions of the mouse auditory cortex in the coronal representation along the vertical gridlines (Fig. 3b) at 0.2-mm intervals between 2.0 mm and 4.0 mm posterior to the bregma, on consecutive Nissl-stained template coronal slices (Figs. 4, 5, 6). The dorsoventral width of each region is summarized in Table 2. We also confirmed that the auditory cortex has no significant left-right differences in size in the C57BL/6 strain (Supplemental Fig. S2). To the best of our knowledge, this is the first coronal atlas that portrays multiple regions that were identified physiologically, with physiological annotations, and that bridges the coronal and cortical surface views of the mouse auditory cortex.

## Discussion

### Quantitative surface map of the mouse auditory cortex

The precise portrayal of the mouse auditory cortical surface map presented here (Fig. 3) can easily be compared to the auditory cortical maps constructed in other rodents. Prior to mouse studies, precise, elaborate maps of the auditory cortex had been elucidated in the rat that is widely used in the neuroscience field. Higgins *et al.* elegantly visualized up to seven frequency-organized regions — the AAF, AI, ventral auditory field (VAF), posterior auditory field (PAF), ventral posterior auditory field (VPAF), and rostral/ventral suprarhinal auditory field (rSRAF/cSRAF)^26^ — using Fourier optical imaging^33^,^34^. The accuracy of these maps has been confirmed by gene-expression patterns^35^, thalamocortical tracing analyses^27^,^35^,^36^, and the consistency with previous findings from large-scale, high-resolution, electrode-based analyses^34^,^37^. However, an auditory cortical map in the mouse had not been properly delineated and until recently the necessary anatomical and histological verifications had not been performed. This might be because the individual regions in the mouse auditory cortex are much smaller (<300 μm in diameter) and are therefore more difficult to map. Benefiting from the development of endogenous fluorophore imaging, recent studies have revealed multiple auditory cortical fields in the mouse, with fine-grained sound frequency response topography^5^,^16^. Our representation of the mouse auditory cortical surface (Fig. 3b) is similar to that already elucidated in the rat^26^,^34^,^38^. Moreover, the unspecific region (UR) in the rat auditory cortex^39^ is in the same location as the non-tonotopical DA region in the mouse^4^,^5^,^7^,^30^. The agreement between these published investigations suggests a possibility that regions in the auditory cortex of the mouse and rat could be homologized in the future.

### Coronal representation of the mouse auditory cortex

The mouse auditory cortex is usually delineated with three major regions in existing coronal atlases, e.g. the primary auditory field, ventral auditory field, and dorsal auditory field (and occasionally the posterior auditory field). These regions are usually illustrated to be laid in parallel rostrocaudally at full length in the auditory cortex. In contrast, flavoprotein fluorescence imaging revealed at least six auditory cortical regions on the cortical surface; the four frequency-organized regions that travel in various directions and the two localized frequency-unorganized regions (Fig. 3). Our coronal atlas (Figs 4, 5, 6) clearly represents dorsoventral positional shifts and the emergence and disappearance of each region in a rostrocaudal direction. This surface-guided mapping of the auditory cortex will compensate for the lack of existing mouse brain databases. In addition, a refinement process of regional segmentation in brain atlases unifies the inconsistent regional nomenclature that is derived from physiological or histological/anatomical studies. The atlas presented here brings physiological nomenclature of the auditory cortex to a mouse brain reference, therefore contributes to establishing experimental environments for comparing physiological findings with vast regional histological properties^40^,^41^.

The coronal representation presented here has several practical limitations. We obtained the coordinate data from C57BL/6 mice which were derived from different litters, therefore our results represent an averaged view of C57BL/6 mice. Hence, it may be difficult to compare our atlas to some genetically-manipulated mice with small brain volumes^42^,^43^ and other mouse strains such as Balb/c. However, this atlas can be used as a reference for intact C57BL/6 mice at wide range of age. We confirmed that the size of the auditory cortex did not change significantly until at least 13 weeks of age (Supplemental Fig. S3). These data are consistent with the previous reports that brain weight is generally constant after the mice become adults, whereas the body weight continues to increase as the mice age^42^,^43^. Because physiological investigations of the sensory cortex are usually conducted using around 10-wk-old C57BL/6 mice, the current atlas is generally suitable for researches using C57BL/6 mice.

Systematic errors that come with histological procedures should also be considered. First, this includes inaccurate rostrocaudal resolution. Brain atlases based on chemoarchitecture^19^ or MRI anatomy^44^, were able to detect segmental guides of the molecular distribution or signal intensities directly on coronal brain sections. In contrast, the template slices in this study contained little information about regional boundaries. Regions were illustrated according to the coordinates on the cortical surface, and the rostrocaudal level was determined according to the Paxinos and Franklin atlas^19^. Therefore we cannot construct an accurate coronal atlas at smaller intervals than does the Paxinos and Franklin atlas. Second, the preparation of coronal sections can generate slice-to-slice variability as slices might be slightly rotated rostrally or caudally by individual researchers. To reduce this variability, we removed the cerebellum in a mouse brain matrix (Stoelting, Wood Dale, IL) to make the brain stand on the pedestal of the cryotome at the same angle for sectioning every time, and the rostrocaudal level was distinguishable at intervals of ~0.2 mm as shown in Figs 4, 5, 6. As the rostrocaudal widths of all the auditory cortical regions are larger than 0.2 mm, this atlas comprehends all the regions. Although technical limitations are unavoidable, our data indicate a small degree of variance between animals for each region (Fig. 3), which indicates relatively small variability in the position of auditory cortical regions and the rhinal fissure ventral to the auditory cortex. More importantly, the size of the auditory cortex established from the constructed map (Fig. 3) and *in vivo* imaging (Supplemental Fig. S2) was almost the same, indicating minimal systematic errors associated with histological procedures, such as slice-to-slice variability and brain shrinkage during perfusion. The present study may therefore represent a foundation for more elaborate databases of the mouse brain.

### Interhemispheric symmetry in the size of the auditory cortex in C57BL/6 mice

Interhemispheric differences in brain structures are prevailing across species. Previous findings have shown interhemispheric asymmetry of the auditory cortex in NMRI mice; the left auditory cortex is larger than the right, particularly along the rostrocaudal axis^45^,^46^. However, significant interhemispheric differences in the rostrocaudal size of the auditory cortex cannot be detected using flavoprotein fluorescence imaging in C57BL/6 mice (Supplemental Fig. S2). In addition, auditory cortical subregions that correspond interhemispherically are located in symmetrical stereotaxic coordinates in C57BL/6 mice^15^ and other mammals^47^,^48^,^49^,^50^,^51^,^52^,^53^,^54^,^55^. These findings suggest that the auditory cortex is symmetrical in terms of the size and bilateral anatomic positioning, at least in the C57BL/6 mouse. This indicates that the stereotaxic information we obtained from the right auditory cortex of the C57BL/6 strain can be applied to the left auditory cortex.

## Methods

### Animals

The experimental procedures in the present study were approved by the Committee for Animal Care at Niigata University. All the experiments were performed in accordance with the approved guidelines and regulations. We used 97 male 6–8-wk-old C57BL/6N mice (Charles River Japan, Kanagawa, Japan). The animals were housed in cages with *ad libitum* access to food pellets and water, and were kept on a 12-h light/dark cycle.

### Functional identification of precise locations of the auditory regions

*In vivo* flavoprotein fluorescence imaging was performed to identify the precise locations of the auditory regions^5^. Mice were deeply anesthetized using urethane (1.65 g/kg, i.p.; Wako, Osaka, Japan), and their rectal temperature was maintained at 37 °C. After local anesthesia using bupivacaine (AstraZeneca, London, UK), the skin and temporal muscle over the right auditory cortex were incised. A piece of metal was attached to the skull with dental resin, and the head was fixed by screwing the metal piece onto a manipulator. The skull over the right auditory cortex was removed. Cortical images (128 × 168 pixels after binning) of endogenous green fluorescence (λ = 500–550 nm) in blue light (λ = 470–490 nm) were recorded using a cooled CCD camera system (AQUACOSMOS with ORCA-R2 camera, Hamamatsu Photonics, Hamamatsu, Japan). The area covered by one pixel was 20.4 × 20.4 μm^2^. Images were taken at 9.7 Hz and averaged over 20 trials. Spatial averaging of 5 × 5 pixels was applied. Images were calculated as ΔF/F_0_, where ΔF = F − F_0._ The baseline intensity (F_0_) was obtained by averaging the intensity values in five frames during the prestimulus period (~500 ms). The response amplitude was evaluated as ΔF/F_0_ for every pixel. To remove the effect of light scatter which is generated when emission light proceeds through the brain parenchyma, the Lucy–Richardson deconvolution was applied to tonal response images where the width of a Gaussian was 200 μm^16^.

Tones were made by a computer using a custom-written LabVIEW program (National Instruments, Austin, TX) at a sampling rate of 500 kHz. Sounds were low-pass filtered at 150 kHz (3624, NF, Kanagawa, Japan). Pure tones at frequencies of 5–60 kHz were amplitude modulated by a 20-Hz sine wave. A speaker for 5 and 30 kHz (SRS-3050A, Stax, Saitama, Japan) or 60 kHz (ES105A, Murata, Kyoto, Japan) was set 10 cm in front of the mice. Sound intensity was calibrated using the microphone (Type 4135 and Type 2669, Brüel & Kjær, Nærum, Denmark) and the sound level meter (Type 2610, Brüel & Kjær). The sound duration was 500 ms with a rise/fall time of 10 ms. The desired sound spectrum was confirmed using a digital spectrum analyzer (R9211A, Advantest, Tokyo, Japan) or a custom-written LabVIEW program. When regions of the DA and DP were specifically activated, FM direction reversal stimulus between 5 and 11 kHz was used^5^,^7^,^30^. The sound intensity used in the present study was set at ~60 dB SPL.

### Visualization of identified regions

To identify auditory regions visualized using flavoprotein fluorescence imaging on coronal brain sections, BDA was injected into the center of each identified regions^8^. A glass capillary (tip diameter ~3 μm) filled with a BDA (molecular weight, 3,000; Molecular Probes, Eugene, OR) solution (0.5% in phosphate buffer) and a platinum wire was introduced into the center of the region in the right auditory cortex, to ~500 μm below the surface. The BDA solution was injected iontophoretically by a 4 μA pulse current (7 s on, 7 s off) for 10 min. BDA was injected into one site per animal. Seven days after BDA injection, mice were deeply anesthetized with pentobarbital (1.0 g/kg, i.p.; Kyoritsu, Tokyo, Japan), and the brains were dissected and immersed in 4% paraformaldehyde overnight. Brains were immersed in 20% and 30% sucrose for one day each. The cerebellum was removed coronally in a mouse brain matrix (Stoelting, Wood Dale, IL), and 40 μm thick coronal sections were cut consecutively using a sliding cryotome (REM-710, Yamato-Koki, Saitama, Japan).

To visualize BDA, sections were initially rinsed in 20 mM phosphate buffered saline (PBS) and incubated in PBS containing 3% hydrogen peroxide (Wako) and 0.1% Triton X-100 for 15 min at room temperature. After rinsing in 20 mM PBS containing 0.1% Triton X-100 (PBST), the sections were incubated for 40 min in 20 mM PBST containing avidin-biotin peroxidase complex (Vectastain ABC kit, Vector Laboratories, Burlingame, CA). Sections were rinsed in 20 mM PBS, and BDA was visualized in a solution comprising 0.05% diaminobenzidine tetrahydrochloride (DAB, Dojindo, Kumamoto, Japan) and 0.003% hydrogen peroxide in 50 mM Tris-HCl buffer (pH 7.4) for 20 min. Sections were finally rinsed in 50 mM Tris-HCl buffer and mounted onto gelatin-coated slides. After the mounted sections had dried, they were dehydrated through a graded ethanol series and cleared in xylene. Sections were counterstained using 0.1% cresyl violet (Chroma Gesellschaft, Kongen, Germany), and were cover-slipped using the covering reagent Bioleit (Okenshoji, Tokyo, Japan). Sections were observed under the light microscope (Eclipse Ni, Nikon, Tokyo, Japan) and imaged using a CCD camera (DP80, Olympus, Tokyo, Japan).

### Outlining the tonal responses obtained using flavoprotein fluorescence imaging

The pixels around a target region were trimmed from an image of tonal responses. The trimmed image was converted into binary with a threshold of >60% of the peak amplitude in the region. Here, isolated pixels which did not abut the largest responsive island were considered as zero. The pixel density of the image was increased 2,500 times (horizontally 50 times, vertically 50 times), and the outline of the responsive area was obtained by applying a Sobel filter^56^. The 3 × 3 kernel of [1 2 1; 0 0 0; −1 −2 −1] or [1 2 1; 0 0 0; −1 −2 −1]′ was convolved with the image for the horizontal or vertical direction respectively, and values in the center pixel of the matrix were obtained as their root mean square. A center coordinate of an outline was obtained by averaging the coordinates of all the pixels composing the outlines. The distances between the center coordinate and each pixel composing the outline were calculated at intervals of 0.05°. All the distance values were averaged by angle across animals. Finally, the number of points was decreased 1/100, by averaging the points within every 5° angle, to smoothen the outlines.

The responsive areas to 5- and 30-kHz tones in the AAF, AI, and AII (or the 30- and 60-kHz areas in the DM) are slightly separated; therefore we systematically connected the low- and high-frequency areas in the intermediate four frequency-organized regions. Frequency organizations in the mouse auditory cortex travel roughly straight^5^,^16^, therefore we plotted additional four points (circular points) between the square plots indicating the injection sites of 5- and 30-kHz areas (or the 30- and 60-kHz areas) equidistantly apart. The additional outlines were calculated so that they changed in shape gradually from the outline of the 5-kHz area towards that of the 30-kHz area (or from the 30-kHz area towards the 60-kHz area) (Fig. 3a) in proportion to the distance, and the whole regional outline was delineated by combining the six small outlines (Fig. 3b).

### Illustrating auditory regions on the coronal template slices

Auditory regions were delineated on Nissl-stained coronal template slices, along the vertical gridlines shown in Fig. 3b, at 0.2-mm intervals between 2.0 mm and 4.0 mm posterior to the bregma. The template slices were prepared from a 7-wk-old mouse. The size of the template was adjusted to the average size of three mice. Regional boundaries were drawn perpendicularly to the cortical surface, so that the boundary lines penetrate ventral and dorsal points marked according to the coordinate information shown in Table 2. Regions were colored from the cortical surface to the edge between layer VI and the white matter. The drawings and images were prepared using CellSense (Olympus), CorelDRAW (Corel, Tokyo, Japan), Adobe Illustrator (Adobe Systems, San Jose, CA), and Adobe Photoshop (Adobe Systems) software.

## Additional Information

**How to cite this article**: Tsukano, H. *et al.* Quantitative map of multiple auditory cortical regions with a stereotaxic fine-scale atlas of the mouse brain. *Sci. Rep.* **6**, 22315; doi: 10.1038/srep22315 (2016).

## Supplementary Material

Supplementary Information

## Figures and Tables

**Table 1:** Positions of the injection sites.

	AAF		AI		AII		DA	DM		DP
	low	high	low	high	low	high		low	high	
R–C axis	2.38 ± 0.05	2.72 ± 0.08	3.58 ± 0.03	3.17 ± 0.06	3.12 ± 0.02	3.13 ± 0.02	2.70 ± 0.09	3.13 ± 0.14	2.89 ± 0.03	3.37 ± 0.12
D–V axis	1.16 ± 0.08	0.67 ± 0.05	1.29 ± 0.14	1.06 ± 0.05	0.71 ± 0.13	0.32 ± 0.10	1.46 ± 0.13	1.58 ± 0.03	1.92 ± 0.17	1.95 ± 0.12
N	5	4	5	5	4	5	6	3	4	5

Coordinates of the injection sites. Values indicate rostrocaudal (R-C) distances from the bregma (up), dorsoventral (D-V) distances from the rhinal fissure (middle), and number of samples (bottom). The values are rounded off to two decimal place. Data are presented as mean ± SEM. N, number of samples. Unit, mm.

**Table 2:** Dorsoventral width of the regions by rostrocaudal position.

	AAF		AI		AII		DA		DM		DP	
	ventral	dorsal	ventral	dorsal	ventral	dorsal	ventral	dorsal	ventral	dorsal	ventral	dorsal
2.0 mm	–	–	–	–	–	–	–	–	–	–	–	–
2.2 mm	1.09	1.27	–	–	–	–	–	–	–	–	–	–
2.4 mm	0.78	1.39	–	–	–	–	1.58	1.63	–	–	–	–
2.6 mm	0.52	1.16	–	–	–	–	1.08	1.90	1.96	2.10	–	–
2.8 mm	0.48	0.89	–	–	–	–	1.03	1.85	1.70	2.19	–	–
3.0 mm	–	–	0.94	1.22	0.19	0.89	–	–	1.45	2.14	–	–
3.2 mm	–	–	0.85	1.36	0.12	0.90	–	–	1.33	1.88	1.83	2.20
3.4 mm	–	–	0.93	1.46	–	–	–	–	–	–	1.71	2.19
3.6 mm	–	–	1.04	1.44	–	–	–	–	–	–	1.74	1.86
3.8 mm	–	–	–	–	–	–	–	–	–	–	–	–
4.0 mm	–	–	–	–	–	–	–	–	–	–	–	–

Dorsoventral edges of each region in a series of rostrocaudal positions. The values indicate coordinates of intersection points of regional boundaries and gridlines in Fig. 3b. The values are rounded off to two decimal place. Unit, mm.
