# Supplementary material for: Quantitative map of multiple auditory cortical regions with a stereotaxic fine-scale atlas of the mouse brain
Source: Sci Rep. 2016 Feb 29;6:22315. doi: 10.1038/srep22315 (PMC4770424; doi:10.1038/srep22315)
Supplement: Supplementary Information [file srep22315-s1.pdf]

Supplemental Information

Quantitative map of multiple auditory cortical regions with a stereotaxic fine-scale atlas of the mouse brain

Hiroaki Tsukano[1,*], Masao Horie[2], Ryuichi Hishida[1], Kuniyuki Takahashi[3], Hirohide Takebayashi[2], Katsuei Shibuki[1]

[1.] Department of Neurophysiology, Brain Research Institute, Niigata University, 1-757 Asahimachi-dori, Chuo-ku, Niigata 951-8585, Japan

[2.] Division of Neurobiology and Anatomy, Graduate School of Medicine and Dental Sciences, Niigata University, 1-757 Asahimachi-dori, Chuo-ku, Niigata 951-8510, Japan

[3.] Department of Otolaryngology, Graduate School of Medicine and Dental Sciences, Niigata University, 1-757 Asahimachi-dori, Chuo-ku, Niigata 951-8510, Japan

*To whom correspondence should be addressed

Additional Methods

We used 26 male 7-wk-old C57BL/6N mice and five male 13-wk-old C57BL/6N mice in the additional experiments.

Size of the auditory cortex was evaluated as rostrocaudal and dorsoventral length using tonal responses revealed by flavoprotein fluorescence imaging. The low frequency areas of the anterior auditory field (AAF) and primary auditory cortex (AI) are located at both ends along the rostrocaudal axis (indicated by blue broken circles in Supplemental Fig. S2a), and the high frequency areas in the dorsomedial field (DM) and secondary auditory field (AII) are located at both ends along the dorsoventral axis[1] indicated by red broken circles in Supplemental Fig. S2a). Therefore, the rostrocaudal or dorsoventral length of the auditory cortex was evaluated using tonal responses a 5 kHz or 60 kHz tone respectively, which is nearly the lowest or highest limit of the mouse hearing range[2,3].

Length was evaluated using distances between lateral edges of diagonal regions. To obtain these values, the images were converted into binary with threshold of >60% of the response amplitude. Images were rotated so as to make the line crossing peak pixels in the AAF and AI horizontally, or that crossing peak pixels in the AII and DM vertically. Rotated mages were cropped in the same spatial resolution as the original images. Using these reconstructed images, distance between the rostral tip of the AAF and the caudal tip of the AI, or that between the dorsal tip of the AII and the ventral tip of the DM was measured. Images processing was conducted using MATLAB (MathWorks, Natick, MA). The Mann-Whitney U-test was used to evaluate statistical differences between two groups using SPSS (IBM, Armonk, NY).

Tones amplitude modulated at 20 Hz were presented via a speaker for 5 kHz (SRS-3050A, Stax, Saitama, Japan) or 60 kHz (ES105A, Murata, Kyoto, Japan). The sound duration was 500 ms. The rise/fall time was 10 ms. The sound intensity was set at ~60 dB SPL.

Additional Discussion

It is generally accepted that the auditory cortex is a brain region where functional and anatomical laterality is conspicuous. In humans, the auditory cortex is located in the Heschl's gyrus and many reports have shown that the left Heschl's gyrus is larger than the right[4,5,6]. The language processing center is generally located in the left hemisphere of the human brain[7], therefore the left hemisphere has a larger area for complex processing of language-related sounds. This unilateral coexistence of advantages in size and communication processing has also been reported in mice. Several studies have found interhemispheric laterality in the auditory cortex of NMRI mice. In these studies, communication calls were processed in the left auditory cortex of NMRI mice[8,9], and the left auditory cortex was found to be ~500 μm larger than the right. This difference was determined by observing the spatial distribution of the best frequencies in both hemispheres[10].

However, our previous study has implied that the two sides of the auditory cortex are symmetrical in C57BL/6 mice; auditory cortical subregions that correspond interhemispherically are located along symmetrical stereotaxic coordinates in the C57BL/6 mouse brain[11]. We have confirmed in the current study that no significant

interhemispheric differences exist in the size of the auditory cortex of C57BL/6 mice. In contrast, previous studies clearly revealed that C57BL/6 mice have left-right differences in the pattern of neural activities or function in several auditory cortical regions[12,13,14]. Therefore, it is likely that the functional laterality in C57BL/6 mice is not based on macroscopic structural differences but on microscopic mechanisms such as neural circuits or the level of receptor expression. This is supported by the recent finding that pup-retrieval in response to communication calls depends upon dominant oxytocin receptor expression in the left auditory cortex[14]. Furthermore, interhemispheric symmetry in the size of the auditory cortex is consistent with recent findings in humans that the volume of gray matter in the auditory cortex *per se* is symmetrical, although the volume of the Heshel's gyrus is asymmetrical[4,5,6,15]. In addition, little evidence has been found that the auditory cortex of cats and monkeys is asymmetrical in size, although functional asymmetry exists[16]. Elucidating the inconsistency in interhemispheric size between mouse species may reveal the mechanism of communication processing in the auditory cortex, because the genes to generate laterality in the size of the auditory cortex could be identified thanks to a mouse advantage of genetic tractability.

References

1. Tsukano, H. et al. Delineation of a frequency-organized region isolated from the mouse primary auditory cortex. *J. Neurophysiol.* **113**, 2900–2920 (2015).
2. Guo, W. et al. Robustness of cortical topography across fields, laminae, anesthetic states, and neurophysiological signal types. *J. Neurosci.* **32**, 9159–9172 (2012).

3. Issa, J.B. et al. Multiscale optical Ca2+ imaging of tonal organization in mouse auditory cortex. *Neuron* **83**, 944–959 (2014).

4. Penhune, V.B., Zatorre, R.J., MacDonald, J.D. & Evans, A.C. Interhemispheric anatomical differences in human primary auditory cortex: probabilistic mapping and volume measurement from magnetic resonance scans. *Cereb. Cortex* **6**, 661–672 (1996).

5. Zatorre, R.J., Belin, P. & Penhune, V.B. Structure and function of auditory cortex: music and speech. *Trends Cogn. Sci.* **6**, 37–46 (2002).

6. Sigalovsky, I.S., Fischl, B. & Melcher, J.R. Mapping an intrinsic MR property of gray matter in auditory cortex of living humans: a possible marker for primary cortex and hemispheric differences. *Neuroimage* **32**, 1524–1537 (2006).

7. Wallentin, M., Michaelsen, J.L., Rynne, I. & Nielsen, R.H. Lateralized task shift effects in Broca's and Wernicke's regions and in visual word form area are selective for conceptual content and reflect trial history. *Neuroimage* **101**, 276–288 (2014).

8. Ehret, G. Left hemisphere advantage in the mouse brain for recognizing ultrasonic communication calls. *Nature* **325**, 249–251 (1987).

9. Geissler, D.B. & Ehret, G. Auditory perception vs. recognition: representation of complex communication sounds in the mouse auditory cortical fields. *Eur. J. Neurosci.* **19**, 1027–1040 (2004).

10. Stiebler, I., Neulist, R., Fichtel, I. & Ehret, G. The auditory cortex of the house mouse: left-right differences, tonotopic organization and quantitative analysis of frequency representation. *J. Comp. Physiol. A* **181**, 559–571 (1997).

11. Horie, M., Tsukano, H., Takebayashi, H. & Shibuki, K. Specific distribution of non-phosphorylated neurofilaments characterizing each subfield in the mouse

auditory cortex. *Neurosci. Lett.* **606**, 182–187 (2015).

12. Ohshima, S. et al. Cortical depression in the mouse auditory cortex after sound discrim- ination learning. *Neurosci. Res.* **67**, 51–58 (2011).
13. Honma, Y. et al. Auditory cortical areas activated by slow frequency-modulated sounds in mice. *PLoS One* **8**, e68113 (2013).
14. Marlin, B.J., Mitre, M., D'amour, J.A., Chao, M.V. & Froemke, R.C. Oxytocin enables maternal behaviour by balancing cortical inhibition. *Nature* **520**, 499–504 (2015).
15. Smilley, JF. et al. Hemispheric asymmetry of primary auditory cortex and Heschl's gyrus in schizophrenia and nonpsychiatric brains. *Psychiatry Res.* **214**, 435–443 (2013).
16. Poremba A. et al. Species-specific calls evoke asymmetric activity in the monkey's temporal poles. *Nature* **427**, 448–451 (2004).

Additional Figures

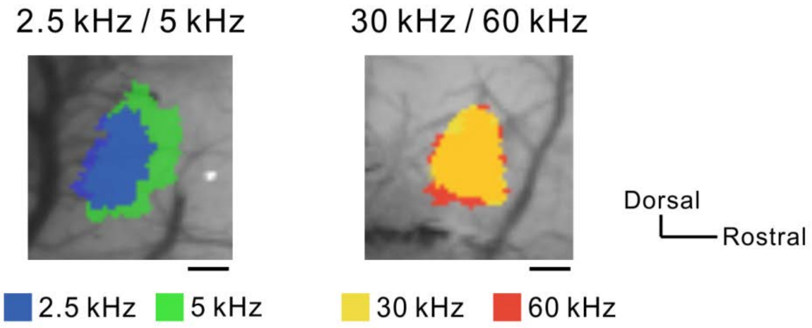

Supplemental Figure 1. Responsive areas elicited by tones <5 kHz and >30 kHz in the AI. Areas responsive to 2.5 kHz and 5 kHz, or 30 kHz and 60 kHz, were almost overlapping, as observed by large-scale optical imaging. Scale bar, 200 μm.

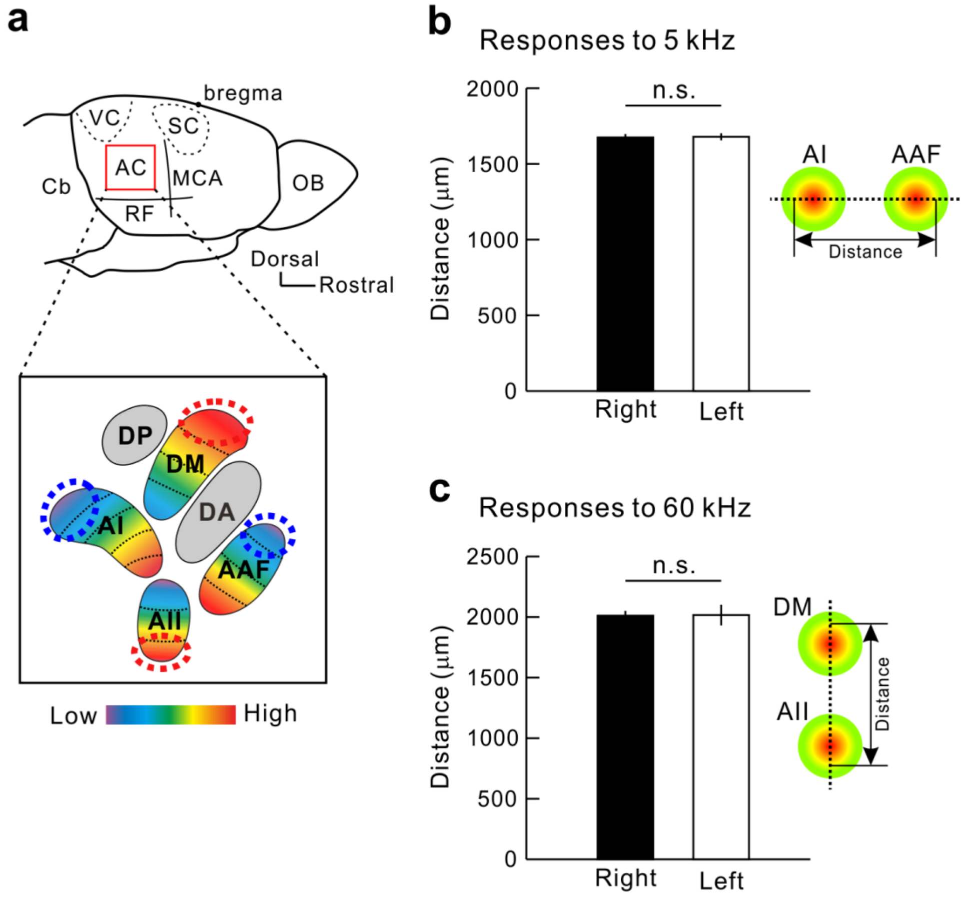

Supplemental Figure 2. Evaluation of the size of the auditory cortex in the left and right hemispheres. (a) The relative positions of the multiple auditory cortical regions. The areas marked by blue broken circles in the AAF and AI represent low-frequency areas and the red broken circles in the AII and DM indicate high-frequency areas (lower panel). These were located in the outer distal parts of the auditory cortex. AC, auditory cortex; Cb, cerebellum; MCA, medial cerebral artery; OB, olfactory bulb; RF, rhinal fissure; SC, somatosensory cortex; VC, visual cortex. (b) Rostrocaudal length of the auditory cortex at 7 weeks of age. Distances between the lateral edges in the AAF and AI were evaluated in the right and left auditory cortices. Right hemisphere, 1,675 ± 23 µm (n = 11); left hemisphere, 1,679 ± 23 µm (n = 13); $p > 0.6$. (c) Dorsoventral length of the auditory cortex at 7 weeks of age. Distances between the lateral edges in the AII

and DM were evaluated in the right and left auditory cortices. Right hemisphere, 2,011 ± 40 μm ($n = 5$); left hemisphere, 2,016 ± 85 μm ($n = 6$); $p > 0.6$. These data indicate no significant bilateral differences in the size of the auditory cortex in C57BL/6 mice.

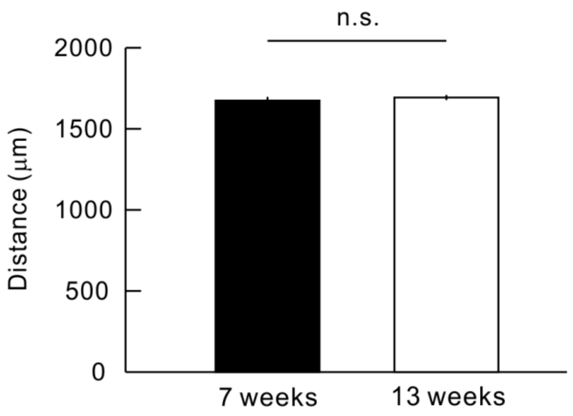

Supplemental Figure 3. Evaluation of the size of the auditory cortex by age. Distances between the lateral edges in the right AAF and AI were evaluated in two different age groups. 7-wk-old mice, 1,675 ± 23 μm (n = 11); 13-wk-old mice, 1,693 ± 17 μm (n = 5); $p > 0.7$.
